# Wild Beasts and Snakes

**Published:** 1897-12-11

**Authors:** 


### Wild Beasts and Snakes.

Few people in England, we imagine, are at all aware of the very heavy mortality which occurs in tropical countries from the attacks of snakes and wild animals. From Government returns issued in India we find that during the year 1896 no less than 24,335 deaths were so caused in that country.

So far as human beings are concerned, by far the largest part of this mortality is due to snakes, and these figures should lead to every encouragement being given to the researches which are at present being carried out by such men as Dr. Calmette and Professor Fraser, with the object of discovering an antidote.

Dr. Calmette has insisted on the identity of the physiological effects of the poisons secreted by the different species of snakes, and has also shown that the serum of animals vaccinated against a very virulent venom, such as that of the cobra, is antitoxic to the venom of all kinds of serpents, and even of scorpions.

Professor Fraser has more recently taken up another line of research, which, although as yet in its infancy, seems full of promise. Struck by the fact that venom enough to kill 1,000 animals if injected under the skin might be swallowed without injury, he has sought for the protective substance, and come to the conclusion that this, whatever it is, lies in the bile, and that, although it is present in largest quantity, or greatest activity, in the bile of poisonous snakes it exists in some degree in that of other animals; and he suggests that it may be possible to prepare from bile an antidote to the effects of snake-bite.

